# Unlocking the hepatoprotective potential of the parasitic plant *Orobanche foetida* Poir. aqueous extract against CCl_4_-induced liver injury in rat

**DOI:** 10.3389/fphar.2023.1320062

**Published:** 2024-01-04

**Authors:** Arij Bedoui, Afoua Mufti, Anouar Feriani, Hanene Baccari, Amal Bouallegue, Mohamed Kharrat, Mansour Sobeh, Moez Amri, Zouhaier Abbes

**Affiliations:** ^1^ Carthage University, Field Crop Laboratory, National Institute for Agricultural Research of Tunisia (INRAT), Menzah 1, Tunisia; ^2^ Laboratory of Biotechnology and Biomonitoring of the Environment and Oasis Ecosystems, Faculty of Sciences of Gafsa, University of Gafsa, Gafsa, Tunisia; ^3^ AgroBioSciences Program, College for Agriculture and Environmental Science, Mohammed VI Polytechnic University, Ben Guerir, Morocco

**Keywords:** Orobanche foetida, phenolic compounds, hepatopreventive, antioxidant, molecular docking

## Abstract

In this current study, we explored the preventive capacity of the aqueous extract of *Orobanche foetida* (OF), a root holoparasite, against CCl_4_ prompt hepatotoxicity in rats. LC-MS/MS profiling revealed the existence of 32 compounds belonging to organic acids, benzoic acid derivatives, and hydroxycinnamic acids along with their glycosides and derivatives as well as several flavonoids. *In vitro*, OF demonstrated substantial antioxidant potential at DPPH and ABTS assays. Results showed that the pretreatment with OF for 6 weeks at the doses (25 mg/kg bw) and (50 mg/kg bw) countered the CCl_4_-induced liver injury by restoring liver injuries indicators (ALT, AST, LDH, ALP, GGT and bilirubin), normalizing lipid profile (TC, TG, LDL-C, and HDL-C), as well as, impeding DNA fragmentation. Furthermore, OF blocked the hepatic oxidative stress spurred by CCl_4_ administration through boosting antioxidant enzymes (GSH, CAT, and SOD) responsible of diminishing lipid peroxidation. exhibited an anti-inflammatory effect by downregulating TNF-α and IL-6 levels. OF suppressive effect on proinflammatory cytokines is further exerted by its capacity to modulate the expression of the NF-κB gene. *In silico* investigation revealed that among the 32 identified compounds, vanillic acid glucoside and dihydroxybenzoic acid glucoside have strong and stable bindings with the active sites of three key inflammatory proteins (PARP-1, TNF-α, IL-6), which could highlight the antioxidant and anti-inflammatory capacity of. Overall, this research provides a preliminary pharmacological support for the medicinal applications of *Orobanche foetida* for addressing inflammatory and hepato-pathological conditions.

## 1 Introduction

Parasitic plants are a very distinct group, notorious for their capacity to penetrate the living tissues of another plant (the host) from which they obtain some or all the nutrients needed for their development throughout their life cycle. This group, comprising over 4,750 species from 26 families, makes up 1.6% of the angiosperm population (Nickrent, 2020). These species, often overlooked and dismissed as mere parasites, have played versatile roles in human societies and have been used throughout the world as food, medicine, and in cultural customs (Kuijt, 1969; Büssing, 2000; Těšitel et al., 2021). More importantly, they possess a noteworthy arsenal of bioactive molecules such as alkaloids, flavonoids, terpenoids, and phenolic compounds that may have potential therapeutic applications in the treatment of various diseases (Ben Attia et al., 2020; Gawenda-Kempczyńska et al., 2022; Suaza-Gaviria et al., 2023). As a matter of fact, the two families Orobanchaceae and Loranthaceae are the most recurrently reported and studied for their therapeutic properties among the parasitic plants’ families (Těšitel et al., 2021).

The genus *Orobanche*, the largest of the Orobanchaceae family with over 170 species distributed throughout the world, includes mostly holoparasitic plant species that depends entirely on the host for their growth and survival due to the lack of chlorophyll (Konarska and Chmielewski, 2020). *Orobanche foetida*, very distinct by its vibrant red color and fetid smell, is a root parasite known to be one of the most devastating species in the Mediterranean region (Abbes et al., 2010). In Tunisia, this species poses a growing challenge for various leguminous plant fields (Amri et al., 2019; Amri et al., 2021). Its presence and large spreading into farmers’ fields has been reported as an emerging issue affecting these crops (Nefzi et al., 2016; Trabelsi et al., 2016; Abbes et al., 2020).

Despite their parasitic and damaging nature, several *Orobanche* species were used for centuries in traditional and folk medicine among several ancient civilizations (Shi et al., 2020). Numerous studies have been conducted on them in the effort of foregrounding their vast spectrum of bioactivities and discovering the wealth of their phytochemical compounds. Within this framework, the biological activities of *O. crenata* phytochemical compounds, including anti-inflammatory and antioxidant activities (Abbes et al., 2014; Nada and El-Chaghaby, 2015; Genovese et al., 2020), anticancer activity (Ben Attia et al., 2020; Hegazy et al., 2020) and hepatoprotective activity (Abo-Qotb et al., 2022), have been studied in depth. Meanwhile, the screening for bioactive compounds with interesting therapeutic traits from *O. foetida* is little laid out. A limited number of studies suggest that *O. foetida* might be a reservoir of antioxidant and antibacterial properties and it could be deployed in human nutrition as well as in various commercial and pharmaceutical products (Abbes et al., 2014; Ben Attia et al., 2020).

The aims of our study were to elaborate a preliminary phytochemical analysis of *O. foetida* and to study how well the aqueous extracts of *O. foetida* (OF) could suppress an induced hepatotoxicity. For this matter, our study offered conclusive evidence regarding the potential free radical scavenging capabilities of compounds, along with their antioxidant properties and their ability to protect DNA and hepatic tissues. Moreover, molecular docking techniques were employed to examine the nature of binds formed between several phenolic compounds and some key proteins engaged in the induced hepatotoxicity.

## 2 Material and methods

### 2.1 Chemicals

Chemicals used in conducting mass spectrometry assays were designated as MS-grade, implying a higher purity level suitable for such analyses, while the remaining reagents were classified under analytical reagent grade. LCgrade acetic acid and acetonitrile were obtained from Fluka (Switzerland) and Thermo Fisher (Waltham, MA, USA), respectively. Methanol, employed for the dissolution of samples, was purchased from Panreac (Barcelona, Spain). Double-deionized water was produced using a Milli-Q system (Millipore, Bedford, MA, USA). Standard compounds were supplied by Sigma–Aldrich (St. Louis, MO, USA). Additionally, both CCl_4_ and kits for biochemical assays were obtained from the same supplier, Sigma–Aldrich (St. Louis, MO, USA).

### 2.2 Plant sampling and extraction

In May 2021, *O. foetida* growing on faba bean (*Vicia faba* L.) were gathered from the research station of Beja in Tunisia. The newly cut entire plants were dried under shade in a drying chamber at ambient temperature and active ventilation. The Khurm et al. (2023) method was modified slightly to extract the dry powder from the entire plant (9 g) using 0.25L of water in a Soxhlet system (Buchi, France) during 6 hours. The extract, after being filtered and concentrated using a rotary evaporator at a temperature fixed at 38°C, was stored at 4°C for further analysis.

### 2.3 Phytocontents

The procedure of Mahdi et al. (2023) with slight adjustments was used to assess both total phenolic content (TPC) and total flavonoids content (TFC). To determine the TPC, The Folin-Ciocalteu colorimetric test was employed. The results have been reported as gallic acid equivalent per g dry weight (mg GAE/g DW). The aluminum chloride (AlCl_3_) method was employed to estimate the TFC. The results were presented as mg quercetin equivalents per g dry weight (mg QE/g DW). To estimate the Total tannin contents (TTC), we used the Yao et al. (2023) procedure. The results were expressed as milligrams of catechin equivalent per Gram dry weight (mg CATE/g DW).

### 2.4 LC-MS analysis

HPLC-PDA-MS/MS system, which consists of a Shimadzu Japan system (Tokyo, Japan) coupled with an MS 8050 mass spectrometer equipped with an electrospray ionization (ESI) source, was used to assess OF phytochemical composition, as previously described by Tawfeek et al. (2023). The fragmentation process was carried out using C18 reversed-phase column (Zorbax Eclipse XDBC18, rapid resolution, 4.6 × 150 mm, 3.5 µm, Agilent, Santa Clara, CA, USA). A gradient of water and acetonitrile (ACN) with 0.1% formic acid each was applied, ranging from 5% to 30% ACN over a 60-minuteperiod, at a flow rate of 1 mL/min. Sample injection was automated using the SIL-40C xs autosampler, and the instrument was operated using LC solution software from Shimadzu, Japan. The MS was performed in the negative mode.

### 2.5 Evaluation of *in vitro* activities

#### 2.5.1 DPPH assay

The Mufti et al. (2022) approach was used to test the extract’s capacity to scavenge DPPH. Briefly, 125 µL of DPPH solution was added to 500 µL of extract at gradient concentrations and 375 µL of deionized water. The control tube contained all reagents except the plant extract. The absorbance was measured at 517 nm. The following formula was used to compute the inhibitory activity:

$$\text{Inhibition }\left(\%\right)=\left[\left(1-\text{ As}\right)\;/\text{ Ac}\right]\times 100$$

where: Ac: Absorbance of the control. As: Absorbance of the sample.

#### 2.5.2 ABTS assay

In accordance with the procedure outlined by Hamed et al. (2020), this test was carried out. Firstly, ABTS (7 mM) was incubated with K_2_S_2_O_8_ (2.45 mM) in the dark at 37°C for 12–16 h to obtain ABTS + radical. The solution mixture was provided with distilled water to achieve an absorbance of around 0.70 ± 0.02 at 734 nm. 10 μL of either ascorbic acid (the positive control) or OF samples with different concentrations were blended with 200 µL of a diluted ABTS + solution. The absorbance was measured at 734 nm after 6 min incubation period. The equation used to measure scavenging activity is:

$$\text{Inhibition }\left(\%\right)=\left(1-\;\left(A/\;A0\right)\right)\;*100$$

with (A) the measured absorbance and (A0) the reagent blank reading.

### 2.6 Hepatoprotective activity assays

#### 2.6.1 Animals and treatments

The proposed experiment was carried out on peer male Wistar rats, weighed around 250 g at the beginning of the experience. The rats were purchased from the Central Pharmacy, Tunisia, and housed in the laboratory cages (Faculty of Sciences, Gafsa, Tunisia) under controlled conditions (Temperature: 23°C ± 2°C, Relative Humidity: 55% ± 5%, and 12 h light/dark cycle, fed on a standard chow diet and water *ad libitum*).

#### 2.6.2 Acute toxicity study

Over a 24-h period, and in order to ascertain the safety of *O. foetida* extract, we monitored five groups administered with different doses (5, 10, 25, and 50 mg/kg body weight) and compared their response to non-treated control group.

#### 2.6.3 Experimental design

Following a fortnight period of acclimation and observation, rats were randomly allocated into six main groups (n = 6), in order to ascertain the pretreatment with OF consequences on CCl_4_-intoxicated rats.

Group I (C): Rats received corn oil and fed regular diet *ad libitum* for 6 weeks.

Group II (OF1): Rats were administered OF (25 mg/kg b. w) dissolved in corn oil for 6 weeks.

Group III (OF2): Rats were administered OF (50 mg/kg b. w.) dissolved in corn oil for 6 weeks.

Group (CCl_4_) IV: Rats were injected with 2 mL/kg b. w. dose of carbon tetrachloride (CCl_4_) intraperitoneally (IP) dissolved in corn oil (Taamalli et al., 2020).

Group V (OF1 + CCl_4_): Rats were pre-treated with 25 mg/kg b. w of orally administered at a dose for 6 weeks, and simultaneous treatment with CCl_4_ dissolved in corn oil.

Group VI (OF2 + CCl_4_): Rats received 50 mg/kg b. w of for 6 weeks, and simultaneously they were intraperitoneally injected with CCl_4_ dissolved in corn oil.

The CCl_4_ was administered by gastric gavage twice per week over 6 weeks. A daily pretreatment with OF1 or OF2 was fulfilled 7 days prior to the CCl_4_ exposure and then daily for the duration of the research assay by mean of gastric gavage.

The animals were cervically decapitated following 6 weeks. Plasma was isolated from blood samples via centrifugation for 15 min at 2000 g. For biochemical and molecular research, the plasma and portions of the liver were kept at −20°C. The remaining organs portions were fixed in 10% formalin for the histological analysis.

#### 2.6.4 Lipidic profile

Following the manufacturer guidelines, kits purchased from BIOMAGHREB (Tunisia) were employed to discern the lipidic profile by quantifying plasmatic levels of LDL-C, total cholesterol (TC), triglyceride (TG), and HDL-C by means of spectrophotometric methods.

#### 2.6.5 Biochemical analysis of liver function enzyme

The activities of aspartate aminotransferase (AST), alanine aminotransferase (ALT), glutamyl transferase (GGT), lactate dehydrogenase (LDH), alkaline phosphatase (ALP) and bilirubin were evaluated spectrophotometrically in the plasma samples, according to the manufacturer’s guidelines. The levels of IL-6, and TNF-α in the plasmatic homogenates were measured using the ELISA assay which was carried out using the diagnostic kits from BIOMAGHREB (Tunisia).

#### 2.6.6 Oxidative stress indicators

Superoxide dismutase (SOD) activity was estimated accordingly to Marklund and Marklund (1974), where the results were presented as units (U) per mg of protein. Catalase (CAT) activity was determined based on the method established by Aebi (1984), and its activity was presented as micromoles of H_2_O_2_ broken down per minute per mg of protein. Finally, the activity of glutathione peroxidase (GPx) was measured following the approach reported by Flohé and Günzler (1984), and results were indicated as nanomoles of GSH oxidized per minute per mg of protein.

#### 2.6.7 NF-ƙB gene expression by semi-quantitative RT-PCR

Liver tissue total RNA samples from both the control and experimental groups were isolated using the iScript™ RTqPCR Sample Preparation Reagent (170–8,898, Bio-Rad) according to the manufacturer’s guidelines. The concentration and purity of the RNA were assessed by determining the absorbance A260/A280 ratios with a NanoPhotometer™ (Implen, GmBH) as described by A. Feriani et al. (2020). Subsequently, 2 µg of RNA was reverse-transcribed using superscript reverse transcriptase (Invitrogen, France) with oligo (dT) 12–18 as a primer in a total volume of 20 µL. The real-time cycler conditions included an initial denaturation step at 70°C for 10 min, followed by 26 cycles of denaturation at 94°C for 5 min, annealing at 58°C, and extension at 72°C for 1 min. For NF-ƙB gene amplification, the primers sequences used were as follows (Forward 5′-GCC​GTG​GAG​TAC​GAC​AAC​ATC-3′ and Reverse 5′-TTTGAGAAGAGCTG CCAGCC-3′).

#### 2.6.8 DNA fragmentation

Hepatic tissues’ DNAs from different groups were isolated using the method phenol–chloroform–isoamyl alcohol (25:24:1). Qualitative assessment of genomic DNA damage was performed by electrophoresis of genomic DNA samples on ethidium bromide-stained 0.8% agarose gels according to a previously described method by Feriani et al. (2020). The unaltered and fragmented DNA fractions were visualized using UV light.

#### 2.6.9 Histopathological examinations

The paraffin-embedded fixed hepatic tissues were sliced into 4–6 µm thick pieces for various histopathological colorations. Hematoxylin-Eosin (H-E) staining was applied to elaborate morphological structure. Different images of the liver from each experimental group were captured at a ×200 magnification via a light microscope.

#### 2.6.10 Molecular docking procedure

Molecular docking procedure were performed according to Horchani et al. (2022). Initially, the SDF files of the identified compounds were downloaded from the website https://pubchem.ncbi.nlm.nih.gov/. The ACD (3D Viewer) freeware was used to optimize the correct geometry of these compounds. The three-dimensional (3D) structures of PARP-1 (PDB ID: 7KK6), TNF-α (PDBID: 7JRA), and interleukin 6 (PDB ID: 1ALU) were obtained from the RCSB Protein Data Bank. SPDBV4.10 was used to manipulate and optimize these structures. OpenBabel 2.4.1 software was used to perform format conversion. The AutoDockTools 1.5.6 package was used to generate docking input files and results. Finally, Biovia Discovery Studio 2019 and Pymol (Edu-Pymol version) were used to visualize, analyze interactions and also used to demonstrate two-dimensional (2D) structures.

### 2.7 Statistical methods

The statistical analysis was conducted using Prism 7.01 (GraphPad, San Diego, CA, USA). All data were subjected to one-way analysis of variance (ANOVA) procedures at a significance level of *p* < 0.05. The outcomes were presented as the mean ± SD, and comparisons between treatment means were highlighted using the Tukey *post hoc* test.

## 3 Results

### 3.1 Phytochemical characterization

**FIGURE 1:**
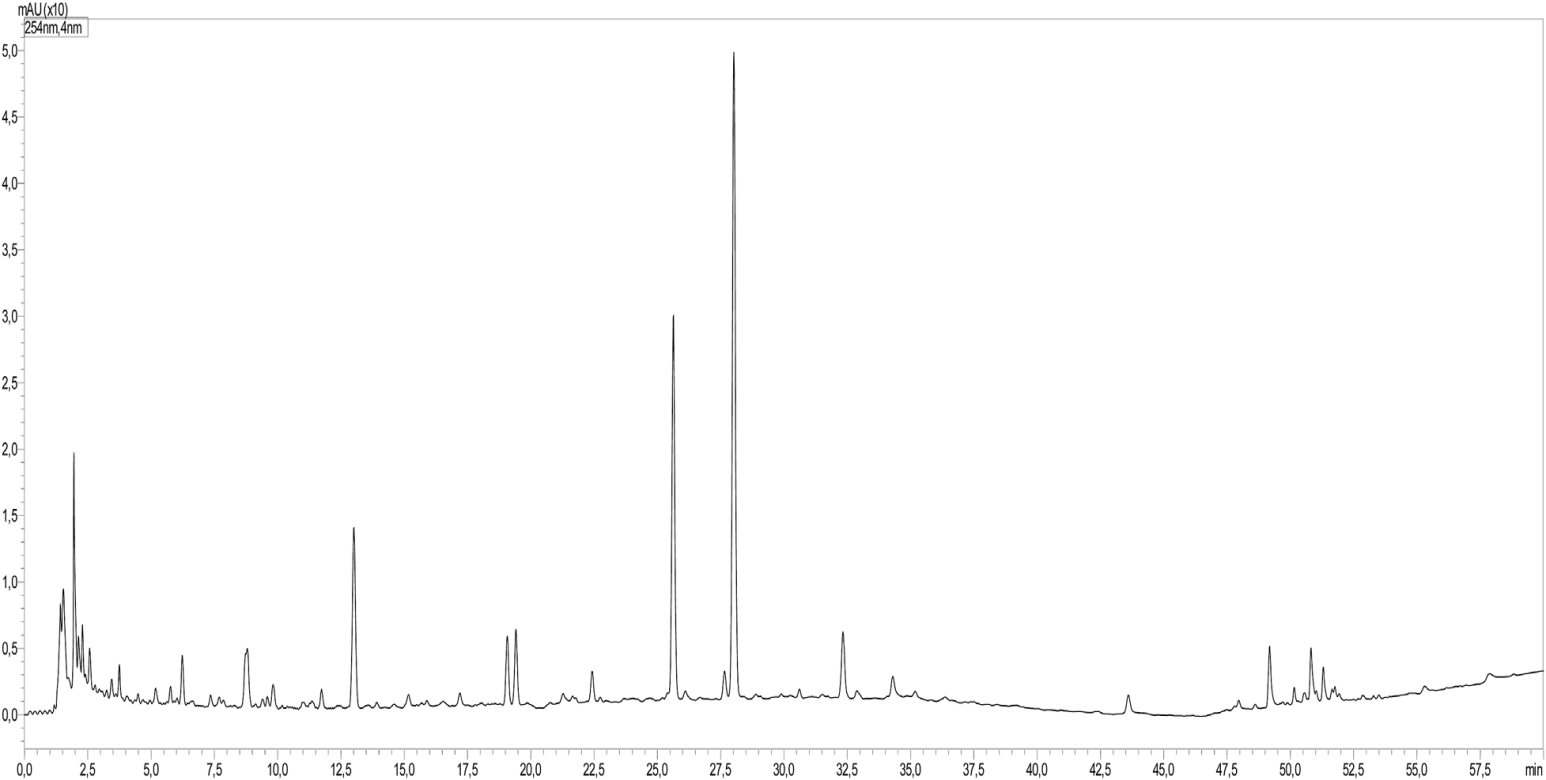
LC-MS profile of *O*. *foetida* extract.

Using HPLC-PDAMS/MS, the identification of peaks in the sample and standards was accomplished by analyzing their MS, MS2 fragments, and retention time. The phytoconstituents analysis revealed the presence of 32 compounds, mainly consisting of phenolic acids and flavonoids (Table 1; Figure 1).

**TABLE 1:** Annotated compounds from *Orobanche foetida* via LC-MS/MS.

Identified compounds	Rt (min)	[M-H]^-^	MS/MS
Malic acid	1.69	133	115
Quinic acid	1.99	191	109
Homovanillic acid malate	2.59	297	181
Vanillic acid glucoside	4.10	329	167
Dihydroxybenzoic acid glucoside	5.24	315	153
Vanillic acid rhamnosyl glucoside	5.76	475	329
Hydroxybenzoic acid glucoside	5.96	299	137
Dihydroxybenzoic acid	6.15	153	109
Hydroxybenzoic acid diglucoside	7.22	461	137
Caffeoyl glucose	7.64	341	179
Coumaroyl glucose	7.71	325	163
Caffeic acid pentosyl glucuronide	8.60	487	179
Caffeoyl glucose	10.53	341	179
Coumaroyl glucose	11.25	325	163
Sinapoyl glucose	11.67	385	223
Caffeic acid	12.87	179	135
Feruloyl caffeoylquinic acid	14.62	529	191
Coumaroyl quinic acid	15.80	337	173
Feruloyl caffeoylquinic acid	17.09	529	179
Quinic acid derivative	18.76	461	191
Caffeic acid derivative	19.19	639	179
Quercetin pentosyl-glucoside	21.23	595	301
Kaempferol rhamnosyl-glucoside	22.73	593	285
Kaempferol pentosyl-glucoside	24.66	579	285
Isorhamnetin pentosyl-glucoside	25.20	609	315
Caffeic acid derivative	25.32	637	179
Isorhamnetin rhamnosyl-glucoside	25.50	623	315
Myricetin rhamnosyl-glucoside	25.68	625	317
Isorhamnetin glucoside	25.92	477	315
Caffeic acid derivative	27.84	621	179
Diosmetin rhamnosyl-glucoside	28.80	607	299
Ferulic acid derivative	32.48	635	193

The identified compounds included various derivatives of benzoic acid, including mono- and di-hydroxy forms, and the corresponding glycosides. In addition, several hydroxy cinnamic acids and their glycosides were identified. The extracts showed the presence of several flavonoids, namely, kaempferol, diosmetin, quercetin, isorhamnetin, and myricetin, and their respective mono- and di-glycosides. Besides, two organic acids, malic acid and quinic acid, were identified.

The Phytocontents dosage and the *in vitro* antioxidant activity results are presented in Table 2. They demonstrate that OF possessed notable polyphenols and flavonoids levels, and it displayed a remarkable antioxidant activity when compared to the standard antioxidant ascorbic acid.

**TABLE 2:** Phytocontents and *in vitro* antioxidant activities of the aqueous extract OF.

	Of	Ascorbic acid
TPC (mg GAE/g extract)	23.13 ± 3.98	-
TFC (mg QE/g extract)	19.31 ± 2.54	-
TTC (mg CATE/g extract)	1.08 ± 0.82	-
DPPH (IC50 mg/mL)	0.71 ± 0.39****	0.56 ± 0.02
ABTS (IC50 mg/mL)	0.68 ± 0.30*	0.72 ± 0.11

Results are given as mean ± SD (n = 6); *p < 0.05 and ****p < 0.0001: OF, vs. ascorbic acid.

### 3.2 Lipid profile studies

Table 3 shows that the rats receiving CCl_4_ showed a dramatic elevation in TC, TG, and LDL-C levels by 129%, 104% and 1,308%, respectively, and a decrease in HDL-C plasmatic level by 57% when compared to control group rats. However, compared to the CCl_4_ Group alone, the pretreatment by OF1 or OF2 restored all the lipid profile indicators. Since the amounts of TC, TG, and LDL-C dropped by 33% 30%, and 48.5%, respectively, for rats treated with OF1 and 25% 40%, and 35% for those treated with OF2. Meanwhile, the assessed level of HDL-C increased by 48% for rats pretreated with OF1 and 61% for rats receiving OF2.

**TABLE 3:** Effect of the aqueous extract of *Orobanche foetida* on lipidic profile.

	C	OF1	OF2	CCl_4_	OF1+CCl_4_	OF2+CCl_4_
TC (mg/dL)	51.51 ± 2.21	54.93 ± 1.97	50.54 ± 3.22	118.00 ± 7.36^****^	79.03 ± 3.60^****^	88.03 ± 5.18^****^
TG (mg/dL)	39.72 ± 2.64	40.58 ± 2.79	39.90 ± 5.01	81.10 ± 2.62^****^	55.99 ± 2.03^****^	48.65 ± 2.03^****^
HDL-C (mg/dL)	24.86 ± 1.93	26.94 ± 2.84	22.38 ± 3.22	10.53 ± 0.84^****^	15.04 ± 0.76^*^	17.02 ± 2.76^***^
LDL-C (mg/dL)	2.79 ± 1.07	3.56 ± 1.47	2.64 ± 0.96	39.59 ± 0.91^****^	20.37 ± 1.02^****^	25.53 ± 0.61^****^

All values are given as mean ± SD (n = 6). *p< 0.05; ***p< 0.001; ****p< 0,0001: control vs. CCl4, CCl4 vs. OF1+CCl4 and CCl4 vs. OF2+CCl4.

### 3.3 Lipid peroxidation analysis

**FIGURE 2:**
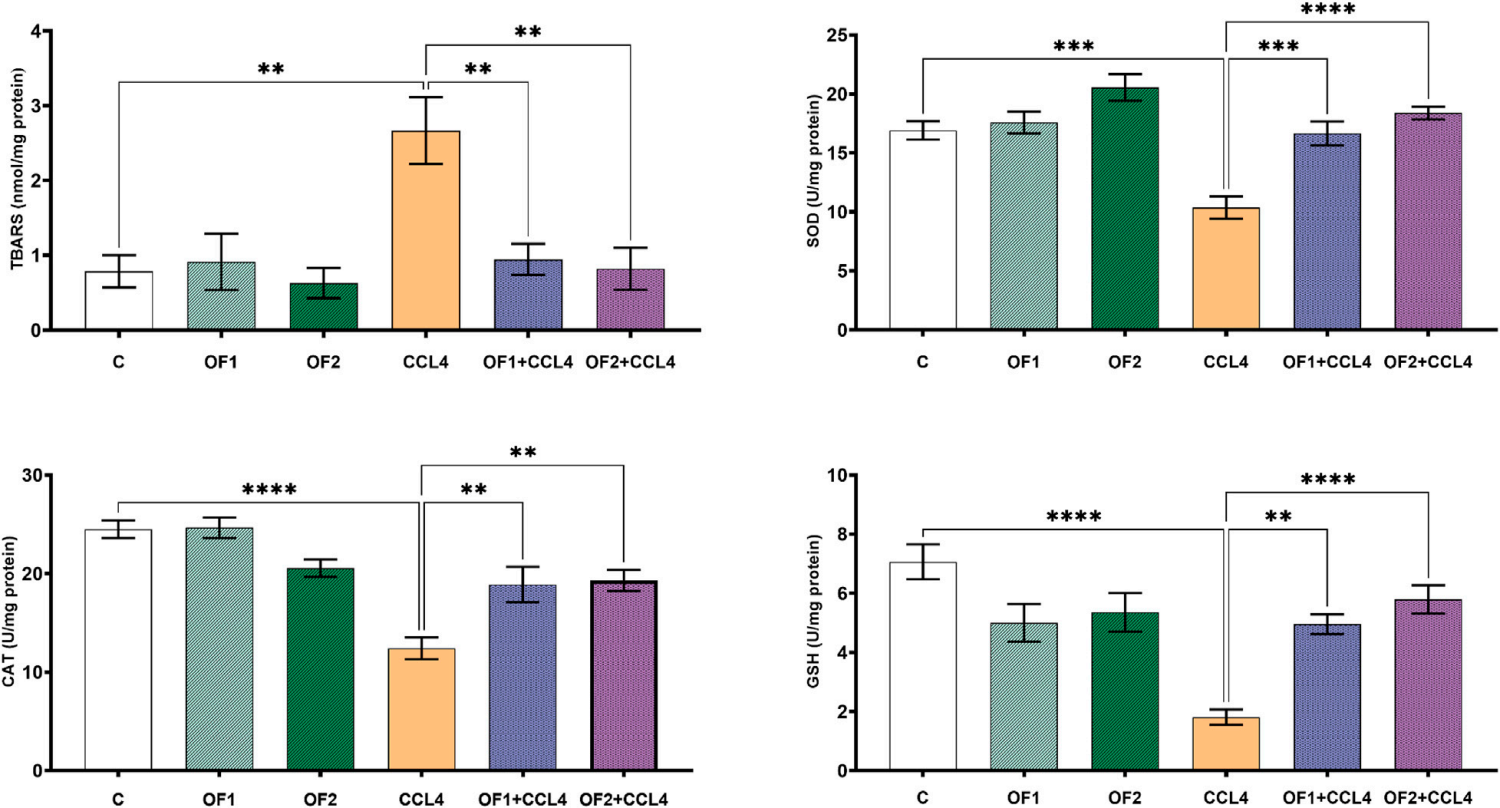
Variation in level of lipids peroxidation and antioxidant biomarkers. Values are given as mean ± sd for groups of six rats **p < 0.01; ***p < 0.001 and ****p < 0.0001: control vs. CCl_4_, CCl_4_ vs. OF1 + CCl_4_ and CCl_4_ vs. OF2 + CCl_4_.

Figure 2 illustrates the amounts of TBARS found in the livers of all experimental animals. Data confirmed that, in contrast to the untreated group, the TBARS’ concentration in the CCl_4_-treated rats soared by 292%. The level of this marker in the liver was recovered after being pre-treated by the two doses OF1 and OF2 prior to the CCl_4_ injection. Given that, when compared to the CCl_4_ group only, the amount of TBARS was reduced to 64% and 69% for (OF1 + CCl_4_) and (OF2+CCl_4_), respectively.

### 3.4 Effects on enzymatic antioxidant levels in the liver

To assess the hepatopreventive activity of, we evaluated its impact on some hepatic oxidative stress markers (SOD, GSH and CAT) after CCl_4_ administration (Figure 2). Aligned with the previous *in vitro* findings, the *in vivo* test results confirmed that the administration of at both doses (OF1 and OF2) mitigates CCl_4_-prompt oxidative stress. Compared to the control group, the hepatotoxicity brought on by CCl_4_ dramatically lowered the activities of SOD, GSH, and CAT in the hepatic tissues by 38% 74%, and 49%, respectively. In comparison to CCl_4_-treated rats alone, pretreatment with the two doses of followed by the injection of CCl_4_ scientifically restored the activities of these enzymatic antioxidants. SOD, GSH, and CAT levels were significantly enhanced by 60%,173%, and 52% respectively, in the (OF1 + CCl_4_) group. And analogously, in the (OF2 + CCl_4_) group, they were increased by 77%, 63% and 55%, respectively.

### 3.5 Effect on the liver injury biomarkers

Table 4 illustrates the outcome of the hepatic injury biomarkers assessment in six groups. The CCl_4_-treated animals had a significant increase in LDH, GGT, ALT, AST, ALP, and Bilirubin activities by 159%, 127%, 214%, 162%, 81% and 303%, respectively, as opposed to the control ones. Yet, compared to CCl_4_-treated groups, the pretreatment of animals with OF1 or OF2 mitigated the impact of CCl_4_ and decreased the level of the marker enzymes LDH, GGT, ALT, AST, ALP, and Bilirubin by 40%, 27%, 34%, 34%, 16% and 20%, respectively, for (OF1 + CCl_4_) group and 38%, 46%, 49%, 30%, 27%, and 43%, respectively, for (OF2 + CCl_4_) group.

**TABLE 4:** Effect of the aqueous extract of *Orobanche foetida* on the hepatic injury marker enzymes.

	C	OF1	OF2	CCl_4_	OF1+CCl_4_	OF2+CCl_4_
ALT **(U/L)**	35.91 ± 3.51	44.16 ± 3.94	40.17 ± 10.08	113.00 ± 60.74^****^	73.86 ± 2.59^****^	57.52 ± 1.53^****^
AST **(U/L)**	62.17 ± 1.41	55.61 ± 3.85	59.30 ± 3.69	163.50 ± 8.90^****^	105.60 ± 7.34^****^	113.60 ± 7.73^****^
LDH**(U/L)**	339.80 ± 5.43	354.50 ± 10.13	333.40 ± 14.97	881.20 ± 8.35^****^	527.30 ± 17.27^****^	540.10 ± 4.09^****^
GGT**(U/L)**	20.40 ± 3.81	19.38 ± 1.96	17.69 ± 2.46	46.48 ± 1.32^****^	33.65 ± 1.11^****^	25.02 ± 2.37^****^
Alp **(U/L)**	48.38 ± 8.14	45.92 ± 5.17	46.15 ± 5.40	87.83 ± 7.66^****^	73.68 ± 4.99^**^	63.68 ± 4.69^****^
Bilirubin **(µmol/L**)	0.13 ± 0.01	0.13 ± 0.03	0.13 ± 0.02	0.53 ± 0.07^****^	0.42 ± 0.06^**^	0.30 ± 0.04^****^

Results were expressed as mean ± SD (n = 6) ****p< 0,0001: control vs. CCl_4_, CCl_4_ vs. OF1+CCl_4_ and CCl_4_ vs. OF2+CCl_4_.

### 3.6 Effects on cytokines levels and NF-κB gene expression

**FIGURE 3:**
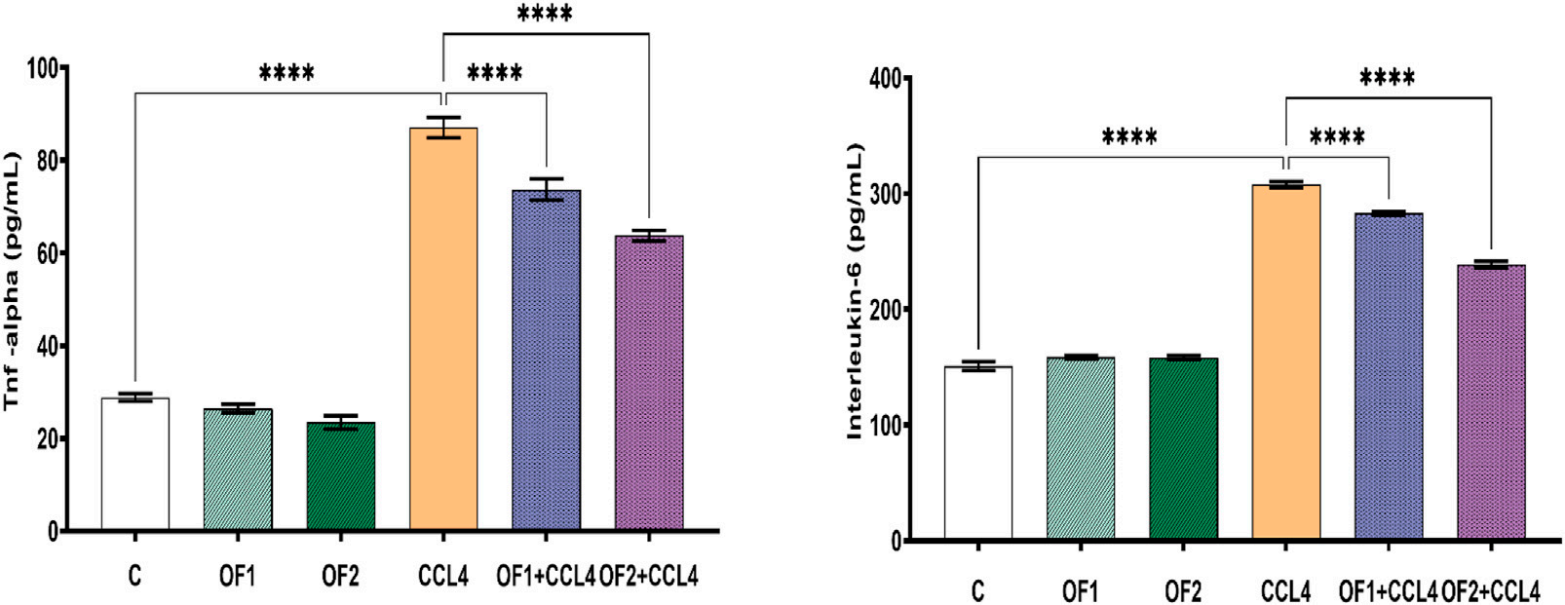
Effect of the pretreatment the aqueous extract of *Orobanche foetida* on IL-6 and TNF-a levels. Values are presented as mean ± sd for groups of six rats ****p < 0.0001: control vs. CCl_4_, CCl_4_ vs. OF1 + CCl_4_ and CCl_4_ vs. OF2 + CCl_4_.

TNF-α and IL-6 are two key pro-inflammatory cytokines. Their evaluation is crucial to confirm hepatic inflammation response (Figure 3). In this study, CCl_4_ is the culprit for a significant rise in both IL-6 and TNF-α around 103% and 202%, respectively. The pre-administration OF1 or OF2 provoked a noteworthy decrease in the elevated TNF-a and IL-6 (*p* < 0.05) compared to the CCl_4_ group. These IL-6 and TNF-α reductions were 8% and 15%, respectively, for the (OF1 + CCl_4_) group and 22% and 26% for the (OF2 + CCl_4_) group.

**FIGURE 4:**
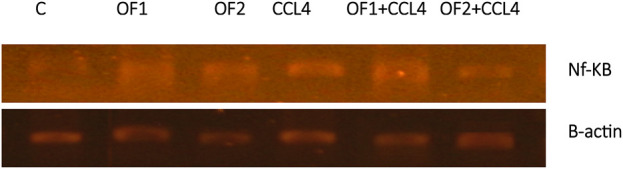
Hepatic gene expression of the nuclear factor kappa B (NF-κB) in control and experimental groups.

Figure 4 depicts the hepatic NF-κB mRNA expression levels in both the control and treated groups. Analysis through semi-quantitative real-time PCR revealed a substantial upregulation of NF-κB mRNA in response to CCl_4_ compared to the control group. Furthermore, the findings indicated that pretreatment with OF1 and OF2 significantly alleviated the expression of NF-κB mRNA compared to the CCl_4_ group. Nevertheless, the mRNA expressions in the extract-treated groups remained unaltered when compared to the control group.

### 3.7 Histopathological findings

**FIGURE 5:**
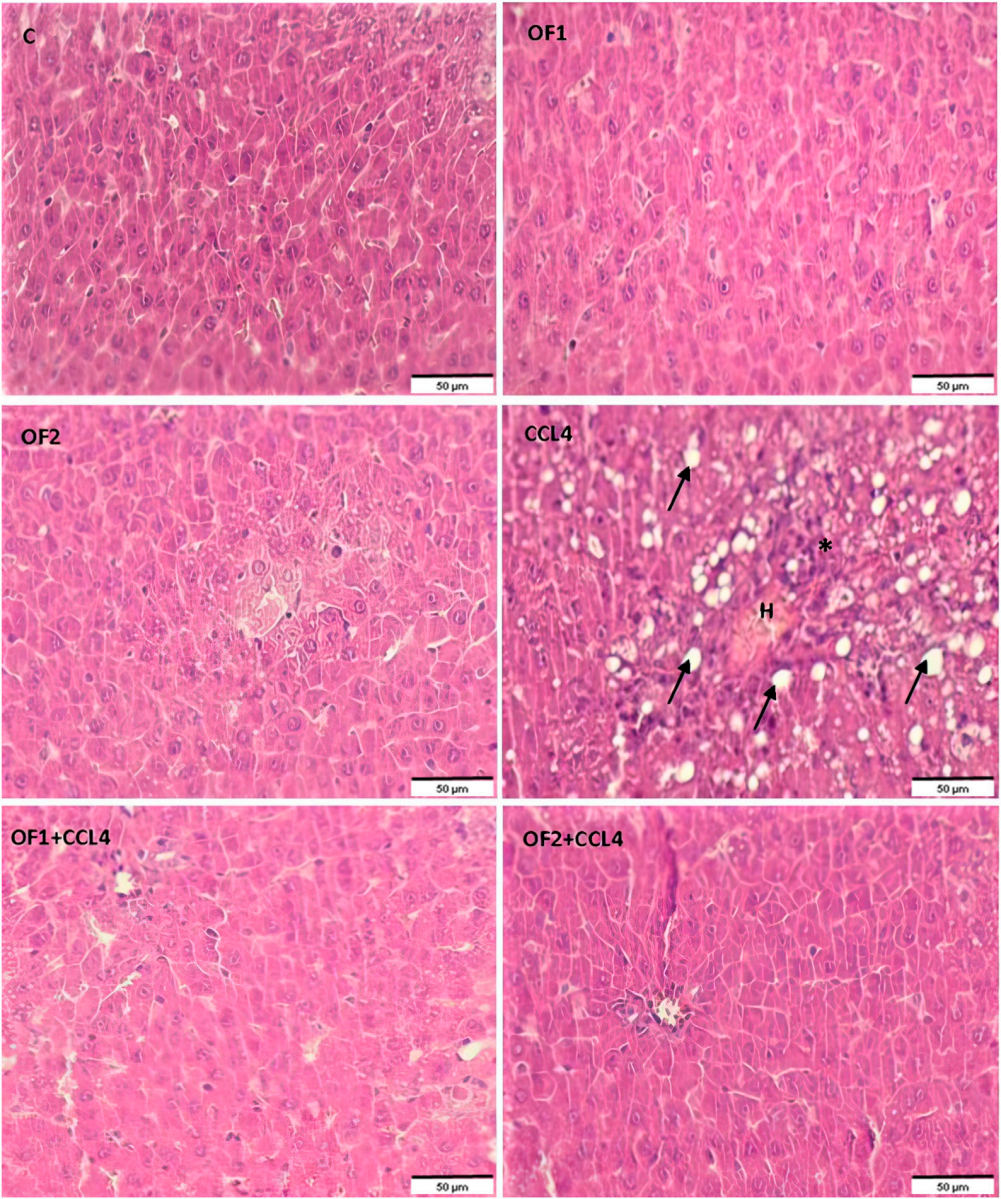
Histological sections of liver of control and experimental treated rats. Liver tissue stained with Hematoxylin and Eosin (G ×200). Asterix: infammatory cell infiltration; arrow: foci of lipid, H: Hemorrhage.

Figure 5 depicts the results of the histological examination using H-E of the control and the treated groups. The control group and the OF1 and OF2 treated groups’ hepatic tissue had a normal structure, with polyhedral hepatocytes arranged around the central vein and spaced by normal sinusoids. The CCl_4_-treated group exhibited dilated sinusoids, excessive congestion of the centrilobular vein, and infiltration of inflammatory cells. The liver injuries that CCl_4_ induced in the rats were greatly reduced by the pre-treatment with OF at both doses.

### 3.8 Evaluation of DNA ladder fragmentation in liver tissue

**FIGURE 6:**
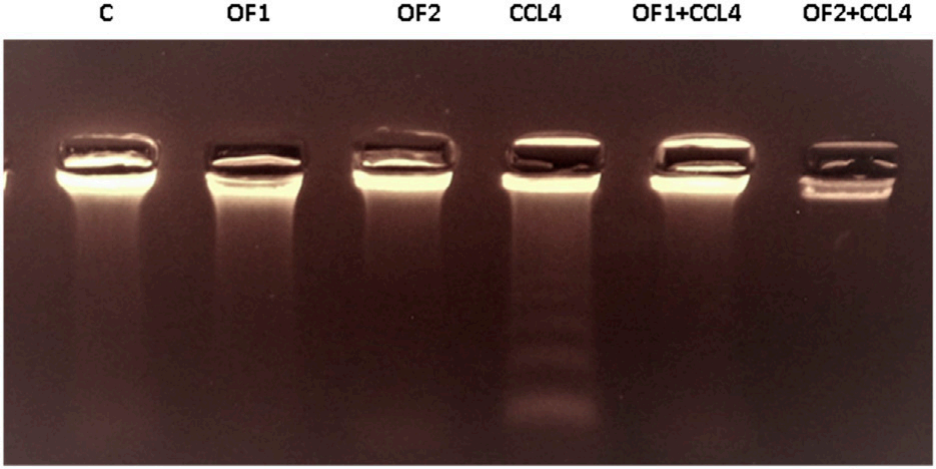
Agarose gel electrophoresis analysis of hepatic DNA from control (Lane 1), OF1 (Lane 2), OF2 (Lane 3); CCl_4_ (Lane 4), OF1 + CCl_4_ (Lane 5) and OF2 + CCl_4_ (Lane 6) treated group.

The variations in the genomic DNA extracted from the liver tissues of the control and experimental groups are shown in Figure 6. An intact band of DNA was visible on an agarose gel electrophoresis of genomic DNA purified from the hepatic tissues of control (lane 1), OF1, and OF2-treated rats (lane 2) and (lane 3), respectively. DNA laddering revealed substantial differences between the CCl_4_-intoxicated group (lane 4) and the control group. Finally, the CCl_4_-induced genotoxicity was reduced by the pre-treatment with OF by the two doses, as evidenced by decreased genomic DNA fragmentation (lane 5) and (lane 6).

### 3.9 Molecular docking inhibition of PARP-1, TNF-α, and IL-6

**FIGURE 7:**
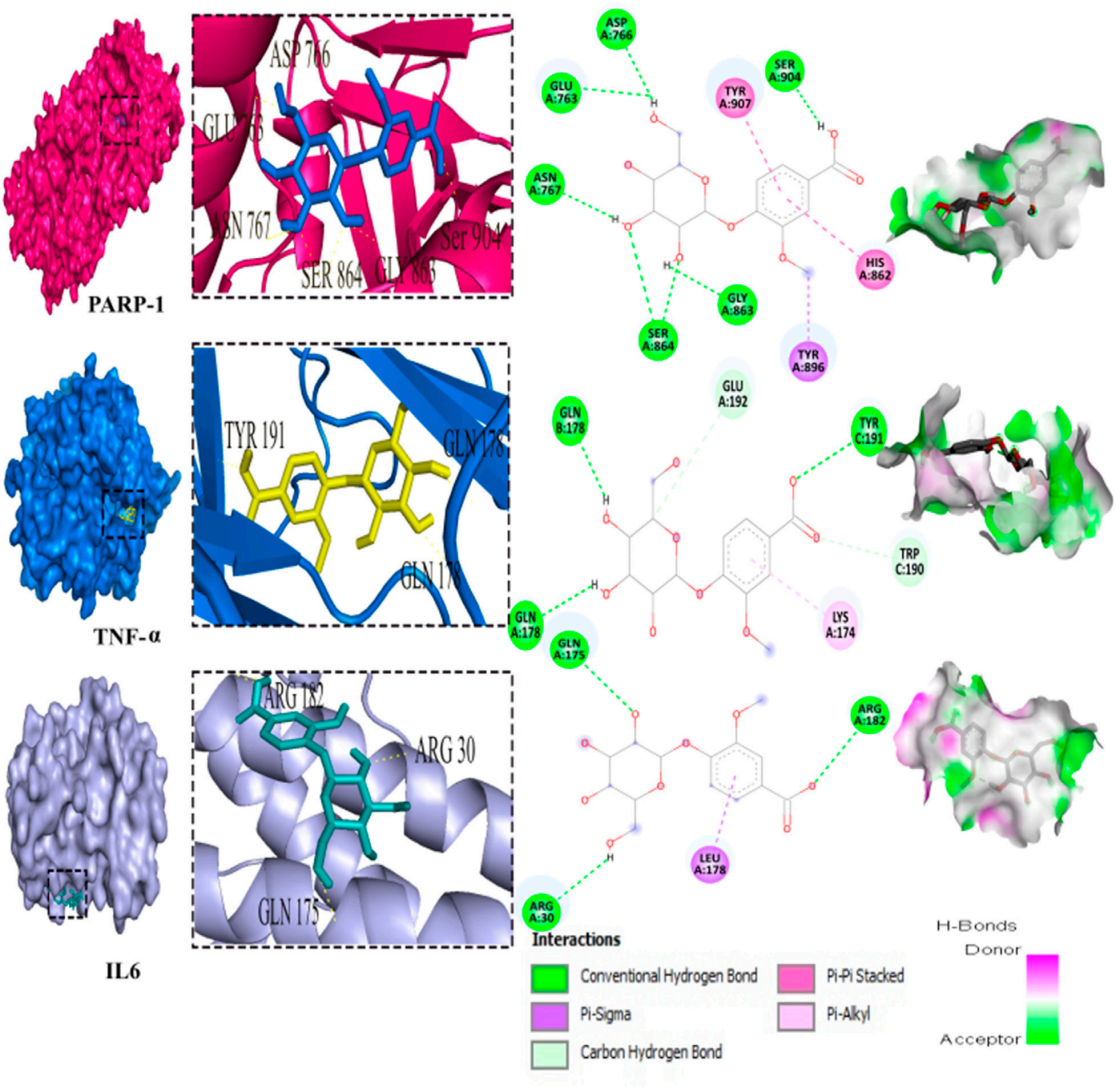
3D representation and 2D diagram of interactions of Vanillic acid glucoside with the best docking scores, bounded to the pocket region of PARP-1, TNF-α and IL-6 with illustration of hydrogen bond.

**FIGURE 8:**
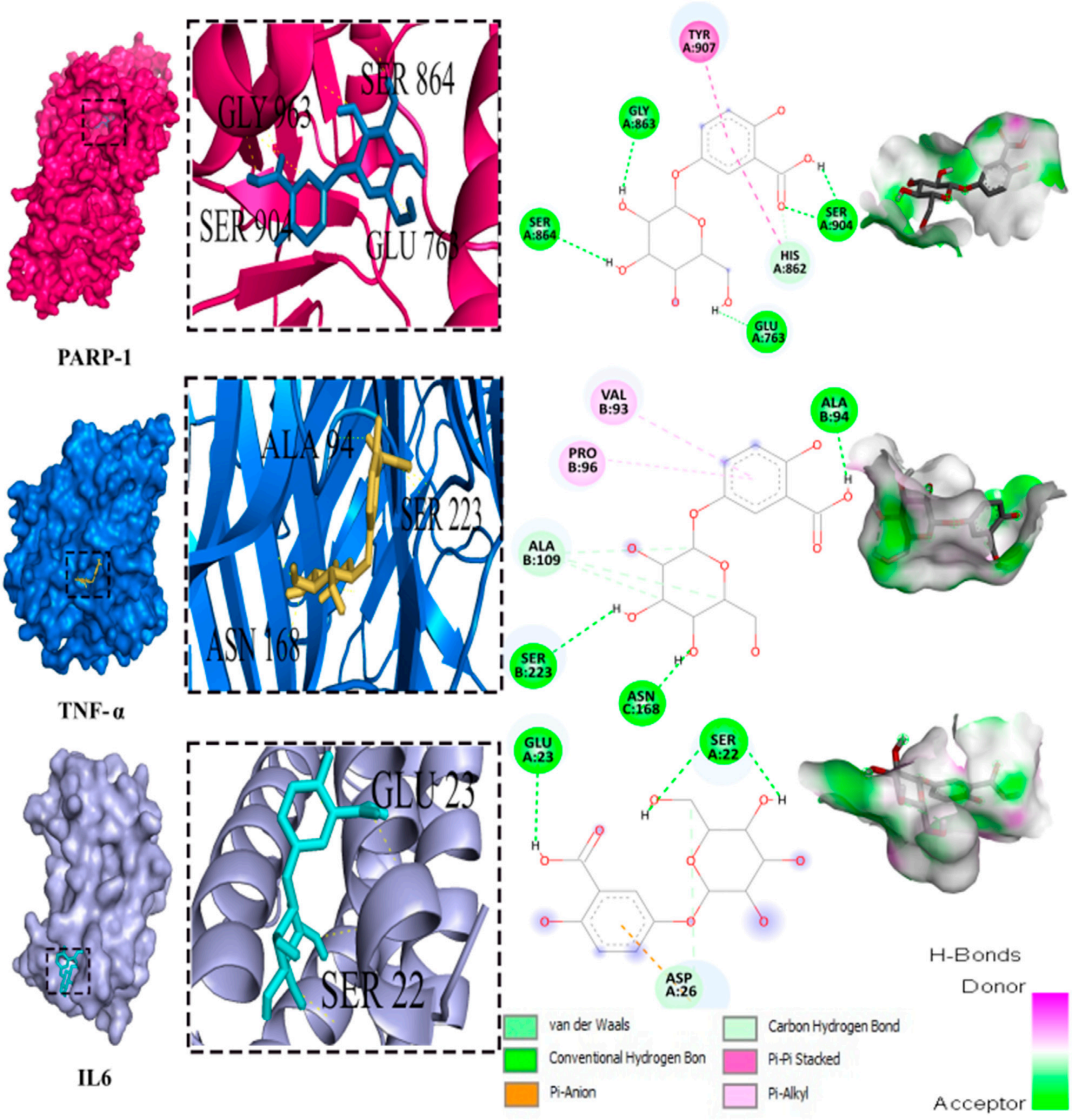
3D representation and 2D diagram of interactions of Dihydroxybenzoic acid glucoside with the best docking scores, bounded to the pocket region of PARP-1, TNF-α and IL-6 with illustration of hydrogen bond.

Out of the 32 compounds identified from the extract, several were excluded due to their molecular weights surpassing 500 Da (Table 1). The remaining compounds underwent molecular docking with PARP-1, TNF-α, and IL-6. The 13 assessed compounds exhibited noteworthy binding energy, as evidenced in (Table 5). Results showed that 12 compounds for both PARP-1 and TNF-α and 10 compounds for IL-6 exhibited free energies lower to −5 kcal/mol. Table 6 summarizes the docking analysis’ outcomes. Besides their favorable binding affinities, several compounds, namely, quinic acid, dihydroxybenzoic acid glucoside, coumaroyl glucose, vanillic acid glucoside, and caffeic acid pentosyl glucuronide, have displayed a multitude of H-bonds surpassing the number of H-bonds formed between the reference drug (Silymarin) and the three enzymes. It is noteworthy that all the compounds have displayed a significant number of H-bonds even with at least one enzyme. Table 6 presents the amino acid residues that could ignite the inhibition of one of three enzymes. This is achieved by highlighting and tracing the specific residues involved in the binding interactions with the reference drug silymarin across all the compounds. Silymarin establishes H-bond with specific amino acid residues within the active sites of the three enzymes, PARP-1, TNF-α, and IL-6: These residues are GLY863, SER864, ASP766, ASP770, ILE879 for PARP-1; PHE140, ASN168 for TNF-α and GLN175 and MET67 for IL-6. We can note that these key residues are recurrently present in several OF compound interactions. In our investigation, we also identified and highlighted some key residues involved in other types of interactions other than H-bond interactions. Vanillic acid glucoside and dihydroxybenzoic acid glucoside, besides not exhibiting any violation of Lipinski’s rules, displayed good energy scores with the three enzymes and formed a significant number of H-bond with several key amino acid residues (Figures 7, 8).

**TABLE 5:** Binding affinities between *Orobanche foetida* compounds and PAR-1, TNF-α and IL-6.

Compounds	Binding affinity (kcal/mol)		
	PARP-1	TNF-α	IL-6
Malic acid	−4.4	−4.6	−4.1
Dihydroxybenzoic acid	−9.1	−6.3	−5.1
Caffeic acid	−7.2	−7.1	−5.1
Quinic acid	−6.2	−6.4	−4.7
Homovanillic acid malate	−6.4	−6.7	−4.9
Dihydroxybenzoic acid glucoside	−8.3	−7.2	−5.7
Coumaroyl glucose	−9.0	−9.3	−5.6
Vanillic acid glucoside	−8.4	−7.6	−5.3
Coumaroyl quinic acid	−6.9	−8.8	−5.8
Caffeoyl glucose	−9.4	−9.3	−5.7
Sinapoyl glucose	−8.9	−8.1	−5.6
Isorhamnetin glucoside	−10.4	−8.6	−5.6
Caffeic acid pentosyl glucuronide	−9.0	−8.5	−5.8
Silymarin^(R)^	−11.7	−8.7	−7.4

(R): reference, drug.

**TABLE 6:** The ligands and PARP-1, TNF-α and IL-6 interactions.

Compounds	Conventional hydrogen bond			Interacting amino acid residues		
	PARP1	TNF-α	IL-6	PARP 1	TNF-α	IL-6
Dihydroxybenzoic acid	3	0	3	TYR907 GLY863 HIS862 SER904^a^ ASP766^a^ ASP770^a^	TYR135 LEU133	LEU62 LEU165^a^ LEU6^a^
						SER169
Caffeic acid	4	5	2	GLU988^a^ TYR907 HIS862^a^ GLY863^a^ SER904^a^	GLY198^a^ ILE134^a^ TYR195^a^ LEU196^a^ SER136^a^ LEU133	ILE136^a^ GLN127^a^
Quinic acid	5	4	6	SER864^a^ GLU763^a^ ASN767^a^ ASN868^a^ ARG878^a^	LEU 196^a^ TYR195^a^ TYR195^a^ TYR195^a^	LEU62^a^ LEU64 LEU64^a^
						PRO65^a^ LEU165^a^ SER169
						GLU172^a^ GLU172^a^
Homovanillic acid malate	4	2	1	GLY863^a^ SER904^a^ SER904^a^ PHE897 TYR896 TYR896^a^	TYR135 LEU133	ASP140^a^
					SER136^a^ LEU196^a^	
Dihydroxybenzoic acid glucoside	5	3	3	TYR907 SER864^a^ GLY863^a^ SER904^a^ SER904^a^ HIS862 GLU763^a^ HIS862	ALA94^a^ VAL93 PRO96 SER223^a^ ASN168^a^	GLU23^a^ SER22^a^
						SER22^a^ ASP26
Coumaroyl glucose	6	3	6	ARG878^a^ ASP770^a^ ASP770^a^ ASN868^a^ ASN767^a^ GLU763 SER864^a^ TYR907 HIS862	TYR195^a^ TYR227^a^ TYR135 LEU133 ILE134^a^	THR82^a^ ASN63^a^ LEU64^a^
						LEU64^a^ GLU93^a^ GLU933^a^ LYS86
Vanillic acid glucoside	7	3	3	ASN767^a^ SER864^a^ SER864^a^ GLY863^a^ TYR896 HIS862 SER904^a^ TYR907 ASP766^a^ GLU763^a^	GLN178^a^ GLU192 TYR191^a^ TRP190 LYS174 GLN178	GLN175^a^ ARG30^a^
						LEU178 ARG182^a^
Coumaroyl quinic acid	2	2	3	TYR896^a^ SER904^a^ GLY863 TYR907 HIS862	LEU133 GLY198^a^ GLY197^a^ LEU233	PHE74 SER76^a^ SER76^a^ GLU172 SER176^a^
Caffeoyl glucose	8	1	3	ASP770^a^ ASP770^a^ ARG87^a^ PHE897^a^ TRP861^a^ SER904^a^ HIS862 TYR907 SER864^a^ ASP766^a^	LEU233^a^ LEU233 LEU133 GLY198	LEU64^a^ LYS66^a^ PRO139 GLU93 GLU93^a^
Sinapoyl glucose	3	3	2	ASP766^a^ ASN767^a^ SER864^a^ TYR907 ARG878 SER904	TYR195^a^ TYR195^a^ TYR227^a^ LEU233 LEU133 ILE231 TYR135	LYS66 SER169 GLU172^a^ SER176^a^ PHE74
						GLU69
Isorhamnetin glucoside	4	4	4	GLY888^a^ TYR896 TYR907 TYR907 ALA898 SER904^a^ HIS862 HIS862 SER864^a^ GLY863^a^ GLU763	GLN178^a^ PRO176^a^ GLN178^a^ GLU192^a^ GLU192 LYS174 TYR191	LYS66^a^ PRO65 LYS86 GLU93 GLU93^a^ GLU93 THR137^a^
Caffeic acid pentosyl glucuronide	5	3	4	SER864^a^ SER864^a^ GLY863^a^ SER904^a^ TRP861^a^ TYR896 TYR907	LEU233^a^ LEU233 LEU133 TYR195^a^ ILE134^a^	LEU33^a^ ASP34
						ARG182^a^ ARG182
Silymarin (ref)	5	2	2	LYS903 GLY863^a^ GLU763 HIS862 SER864^a^ ASN868 ASP766^a^ ASP770^a^ ILE879^a^ GLY876 ARG878	ILE173 PRO193 ALA172 PHE140^a^ ASN168^a^ LEU139	ARG179 GLN175^a^ LEU178 ARG182
						MET67^a^

^a^ Amino acid residue involved in H-bond formation.

## 4 Discussion

This study is one of the few studies that documented and investigated the phytochemical composition of *O. foetida* and its potential use in diseases protective effect. Phenolic compounds are known to be the primary bio-antioxidants present in plants (Kumar and Goel, 2019). In our preliminary analysis, the approximate levels of TPC, TFC and TTC were found to be around 23.13 mg GAE/g DW, 19.31 mg QE/g DW, and 1.08 mg CATE/g DW, respectively. These findings are consistent with the research conducted by Abbes et al. (2014). That further supports our decision to utilize an aqueous extract rather than a methanolic extract, as both extracts demonstrated similar chemical profiles.

The use of chromatographic analysis (LC/MS-MS) enabled the identification of 32 compounds. It is important to note that several peaks observed in the chromatogram remain unidentified, and their identification could potentially provide valuable insights into the phytochemical characterization of this species, particularly if they turn out to be phenylethanoid glycosides. As previous research on phytochemicals from parasitic plants has placed significant emphasis on these phenylethanoid glycosides due to their occurrence exclusively in parasitic plants and their notable antioxidant properties (Kartbaeva et al., 2017; Jedrejek and et al., 2020).

Liver injury is a multifactorial pathology that can arise from several etiological factors, including viral infection, drug overdose, alcohol abuse, and toxicity from exposure to various xenobiotics. Carbon tetrachloride (CCl_4_) is a chlorinated hydrocarbon utilized in several industries. Prolonged or excessive exposure to this substance can induce hepatotoxicity and liver damage. CCl_4_ is metabolized by a group of enzymes known as the cytochrome P450 giving trichloromethyl radical (CCl_3_∗) (Dua et al., 2023). This CCl_3_∗ radical interacts with reactive oxygen species (ROS), leading to the generation of another radical known as trichloromethyl peroxyl radical (OOCCl_3_∗). The presence of the two radicals CCl_3_∗ and OOCCl_3_∗ contributes to an increase in oxidative stress, triggering various pathways including lipid peroxidation (Erdaş et al., 2021), disruption of the glutathione pathway (Bikheet et al., 2022) and the production of pro-inflammatory cytokines, specifically TNF-α (Devi et al., 2021) and IL-6 (Wali et al., 2021). These cumulative effects ultimately contribute to liver damage. Therefore, in experimental models of hepatotoxicity, carbon tetrachloride (CCl_4_)-induced injury is a well-established model that closely mimics the physiopathology of human liver injury (Okoro et al., 2022).

Antioxidants are infamous for playing a critical role in minimizing cellular damage and subsequently apoptosis (Ali et al., 2020; Feriani et al., 2021). we examined the antioxidant potential of using two complementary assays, including assessments of its scavenging activity against DPPH and ABTS. Through these evaluations, we sought to gain a deeper understanding of’s antioxidant properties and its ability to mitigate the damaging effects of oxidative stress on cells. Results confirmed OF’s significant ability to scavenge free radicals, as opposed to ascorbic acid, owing to its bio-antioxidant molecules’ richness, as previously reported by (Genovese et al., 2021).

On the other hand, according to the results of our current study, OF contains secondary metabolites such as tannins, flavonoids, and phenols, as indicated by our phytochemical analyses. It is worth noting that several of these compounds have previously been demonstrated to exhibit antioxidant and hepatoprotective activities (Tir et al., 2019; Feriani et al., 2020; Saadaoui et al., 2023). Hence, the intent was to investigate the potential antioxidant and hepatoprotective effects OF’s phenolic phytochemicals. A possible explanation for these effects is the presence of quinic acid reported to possess antioxidant properties, which may be responsible for the observed reduction in oxidative damage to liver tissue in experimental animals (Lekmine et al., 2020). Furthermore, both the antioxidant and hepatoprotective effects of the extract can also be attributed to the flavonoids present in the extract, especially quercetin. quercetin a well-documented anti-inflammatory and antioxidant flavonoid (Başaran et al., 2022).

*In vivo* experiments showed that the biomarkers (AST, ALT, LDH, ALP, Bilirubin, and GGT) amounts were significantly increased after CCl_4_ treatment, aligned with the previous results of Tlili et al. (2018). As reported by Wali et al. (2021), these biomarkers suggest that liver injury may be due to altered membrane permeability and/or hepatocyte necrosis. Our current study confirmed this hypothesis and demonstrated extensive damage to liver tissue, including necrotic cells, centrilobular venous occlusion, leukocyte infiltration, and Kupffer cell proliferation. These results are aligned with previous studies on *O. crenata* that generated similar findings (Abo-Qotb et al., 2022). The fact that administrating OF significantly decreased high AST, ALT, LDH, ALP, Bilirubin and GGT levels further supports the idea that this extract may be able to shield the liver from the harm that CCl_4_-induced exposure can cause. The capacity of to preserve the integrity of liver cell membranes and enhance liver function may be the origin of this hepatopreventive action. The outcomes of this study revealed a noteworthy reductions of hepatic injury ant pro-oxidative indicators following the pre-treatment with OF prior the exposure to CCl_4_. In addition, taking into account the adverse effects in CCl_4_ group, there was a considerable decrease in antioxidant enzymes consumption. In fact, Hepatocytes have evolved complex defense systems based on different antioxidant enzymes, including CAT, GPx, GST, and GSH, to counteract CCl_4_ damages (Kostic et al., 2022). The excessive production of reactive oxygen species (ROS) might overwhelm antioxidant defense mechanisms in pathological situations like acute CCl_4_ toxicity and result in cellular damage. By triggering the release of proinflammatory cytokines like tumor necrosis factor (TNF-α), interleukin-1 (IL-1), interleukin-6 (IL-6), and nitric oxide (NO), such damage might further accentuate inflammatory responses (Yu et al., 2021). The obtained results showed that pretreatment with OF extract was capable of reducing the oxidative stress state brought on by CCl_4_. To achieve this effect, the OF had to enhance the activities of crucial endogenous antioxidant (SOD, CAT, and GSH) and counters protein oxidation and lipid peroxidation, according to previous studies (Ahmed et al., 2011). According to these results, OF has strong antioxidant properties that may help protect the liver from oxidative stress’s harmful effects. Moreover, caffeic acid present in OF has been previously reported for its ability to increase the endogenous antioxidant enzymes, especially GSH, as well as its ability to decrease the lipid peroxidation in hepatic tissues (Yang et al., 2013).

The study’s findings demonstrated that CCl_4_ exposure led to significant alterations in the lipid profile. However, the alteration of lipid markers brought on by CCl_4_ in the plasma of rats was mitigated by the pre-administration with OF. The inhibitory effects on pancreatic lipase, the most significant enzyme involved in fat digestion, may be the cause of’ hypolipidemic impact. Moreover, the inhibition of HMG-CoA reductase, a crucial enzyme in cholesterol biosynthesis, may be the cause of the observed hypocholesterolemia. This impact has previously been documented in research by (Allagui et al., 2015; Chang et al., 2020). The rise in HDL-C levels due to OF administration is linked to SR-BI receptor’ suppression, which is a widely known HDL receptor that stimulates HDL production, as noted in previous studies (Cariello et al., 2021). Additionally, the decrease in circulating LDL-C levels after OF pre-treatment may be attributed to hepatic LDL-R gene’ safeguarding, resulting in active elimination and uptake of LDL fractions from circulation and ultimately leading to reduced LDL-C in the blood, as documented by Feriani et al. (2020).

The progression of hepatic tissue injury is known to be considerably enhanced by oxidative stress-induced hepatocyte apoptosis, according to the literature (Ghosh et al., 2023). Results demonstrating that CCl_4_ treatment dramatically enhances DNA damage and apoptosis, are consistent with this concept. These findings are aligned with those published by (Maalej et al., 2017; Khan et al., 2019), which showed that administering CCl_4_ causes DNA damage by producing ROS. The current study demonstrates that administering OF within liver tissue decreases DNA fragmentation, suggesting that the pre-treatment with OF reduces oxidative stress-induced apoptosis. This may correlate with the presence of quercetin pentosyl-glucoside, a flavonoid proven to have antioxidant and DNA-protective properties (Ansar et al., 2016).

The histological results showcase that pre-treatment with OF significantly mitigates the occurrence of centrilobular necrosis in animals that had been subjected to the acute toxic effects of CCl_4_, as well as the inflammatory infiltrate. These results suggest that OF might mitigate the liver damage brought on by CCl_4_, possibly through its bioactive components.

The potential hepatopreventive effect of may be attributed to its richness in phenolic compounds, especially flavonoids. Flavonoid-rich plants are well documented to exhibit hepatoprotective effects by inhibiting key enzymes involved in inflammatory processes, such as PARP-1, TNF-α, and IL-6 (Iqbal et al., 2022; Huang and kraus, 2023). Taking account of the pivotal implication of inflammation in hepatic injury prompted by CCl_4_, ROS are not only the source of liver damage, they initiated inflammation by releasing proinflammatory cytokines from activated macrophages (Lin et al., 2012). CCl_4_ activates Kupffer cells to release TNF-α which stimulates the release of IL-1β, and IL-6 that eventually cause hepatic necrosis and alter plasmatic biomarkers (Iqbal et al., 2022; Lu et al., 2015). Besides oxidative stress triggers the expression of NF-κB expediting the synthesis of proinflammatory cytokines. Thereby treatment with OF significantly impedes NF-κB expression, contributing to the inhibition of the inflammatory cascade and ultimately reducing hepatic fibrosis. Previous studies have underscored the capability of bioactive compounds to alleviate the production of pro-inflammatory cytokines by inhibiting the expression of profibrotic mediators such as NF-κB. (Eltahir et al., 2020; Feriani et al., 2020).

As for PARP-1, this enzyme is mainly known for its involvement in DNA damage repair and the regulation of the expression of inflammatory factors. Still, the overactivation of PARP-1, occurring by exposure to CCl_4_ is culprit for the pathophysiological mechanism of hepatic fibrosis. Since it can trigger the activation of hepatic stellate cells HSC that are responsible for liver fibrosis (Huang and kraus, 2023).

Using molecular docking simulations, this work displays the interaction between several phytocompounds found in OF with the binding sites of the three enzymes (PARP-1, TNF-α, and IL-6). The interactions were analyzed to predict their binding mode and to explain their hepatopreventive activity. Ten identified compounds of the extract (Dihydroxybenzoic acid glucoside, vanillic acid glucoside, caffeic acid pentosyl glucuronide, dihydroxybenzoic acid, caffeic acid, coumaroyl quinic acid, caffeoyl glucose, sinapoyl glucose, isorhamnetin glucoside) subjected to molecular docking fit perfectly to the three proteins’ active sites and showed good binding energy scores ranged from −5.0 to −10.4 Kcal/Mol analogously to the reference drug Silymarin. These findings may explain the OF potential hepatopreventive effect highlighted by the previous assays’ outcomes. By comparing the interactions between all the molecular docking studies compounds in the OF and the three enzymes (PARP-1, TNF-alpha, and IL-6) with the interactions of the reference drug silymarin with the previously mentioned proteins, we can note that some specific residues could play a significant role in their inhibition. The residues GLY863, SER864, HIS862, ASP766, and ASP770 for PARP-1, the residues GLN175, ARG182, and LEU178 for IL-6, and the residue ASN168 for TNF-α, were found to be highly involved and represented among several OF compounds. These findings can prove that these residues must be engaged in the inhibition of these inflammation-associated proteins. Subsequently, the interactions between these specific residues and several OF compounds, especially via H-bond, foreground the promising anti-inflammatory and hepatopreventive effect of the extract.

Both vanillic acid glucoside and dihydroxybenzoic acid glucoside stand out as two promising candidates for this hepatopreventive effect through their good binding score, their multiple hydrogen bonds, and their alignment with some key amino residues. Numerous studies have already outlined diverse protective effects of vanillic acid and its derivatives against cancer, diabetes, obesity, neurodegenerative, cardiovascular, and hepatic diseases (Anbalagan et al., 2017; Taqvi et al., 2021; Kaur et al., 2022; Hu et al., 2022) suggested that the supplementation of Vanillic acid glucoside can alleviate oxidative stress and reduce the level of pro-inflammatory cytokines (IL-1β, IL-2, IL-6, and TNF-α).

Every scientific investigation is bounded by certain limitations, and our study is no exception. Firstly, our decision to use only the entire plant of *O. foetida* may have obscured the potential differences and richness in phenolic compounds across different plant parts (stem, flowers, and seeds …) as reported in previous studies. (Khanam et al., 2015; Bitwell et al., 2023). Secondly, Expanding the sample size (n = 6) in our *in vivo* study could enhance the statistical power and address any ambiguity regarding parameter variations. Concerning the histopathological studies, employing additional staining techniques like Sirius red and Masson’s trichrome staining could foreground other anomalies, such as the distribution of total collagen (Peugnet-Gonzále et al., 2023). Besides, in molecular docking studies, by following the Lipinski rule of five, we excluded some powerful bioactive compounds like quercetin, kaempferol, and myricetin due to their high molecular weight. Finally, regarding the clinical relevance to human health, our study, though carried out on an animal model, is a promising preliminary investigation that could ignite considerations for humans uses. To start, we need to address the conundrum of the extrapolation of our results to human uses by recognizing and exploring the interspecific variations specially in hepatoprotection involved pathways and implementing future clinical trials to validate the observed effects in human subjects, bridging the gap between preclinical research and the therapeutic applications of *O*. *foetida*.

## 5 Conclusion

In this study the authors explored the potential antioxidant and hepatopreventive activities of *O. foetida* phytochemical composition via LCMS/MS. Results exhibited a noteworthy level of interesting molecules with potent hepatopreventive and antioxidant activities. *In vitro* and *in vivo* assays have revealed the remarkable antioxidant properties of, as well as, its capacity to mitigate CCI_4_-induced hepatotoxicity by counteracting oxidative stress, DNA fragmentation, and hepatic tissue alterations. Molecular docking analysis underscored the strong and stable binding pattern between two OF compounds: Vanillic acid glucoside and Dihydroxybenzoic acid glucoside with PARP-1, TNF-α, and IL-6, three enzymes infamous for their role in hepatotoxicity and inflammatory response.

Further advanced work is yet needed to isolate the interesting bioactive molecules and to fully grasp the pathways and the mechanisms of action involved in their antioxidant and hepatopreventive activities, for the purpose of developing a new natural, non-hazardous pharmaceutical drug.

## Data Availability

The original contributions presented in the study are included in the article/Supplementary material, further inquiries can be directed to the corresponding authors.
